# Effect of the *rs2590498* (A/G) Polymorphism of the *OBPIIa* Gene on the Olfactory Threshold and Perception Intensity of Fatty Acids

**DOI:** 10.3390/foods15112006

**Published:** 2026-06-04

**Authors:** Daniela Diana, Melania Melis, Iole Tomassini Barbarossa, Roberto Crnjar, Giorgia Sollai

**Affiliations:** Department of Biomedical Sciences, University of Cagliari, 09042 Monserrato, CA, Italy; d.diana6@studenti.unica.it (D.D.); melania.melis@unica.it (M.M.); tomassin@unica.it (I.T.B.); crnjar@unica.it (R.C.)

**Keywords:** gas chromatography-olfactometry technique, n-butanol threshold test, perireceptor space, human olfactory function, interindividual variability, saturated and unsaturated fatty acids

## Abstract

The perception of the odor of fatty acids provides individuals with information about the nutritional content of foods. This perception varies depending on biological and genetic factors. Previous studies have shown that odorant binding proteins (OBPs) present in olfactory mucus play an important role in capturing and transporting odorants, typically lipophilic molecules, through the mucus to the olfactory receptors (ORs). The main objective of this research was to study the role of the *rs2590498* (A/G) polymorphism of the human *OBPIIa* gene on the threshold and intensity of odor perception of palmitic (PA), oleic (OA) and linoleic (LA) acids. Volunteers were genotyped for OBP polymorphisms and classified as normosmic or hyposmic based on their threshold for n-butanol. The results show that normosmic or AA genotype participants perceived the odors of PA, OA, and LA at lower concentrations and with greater intensity than hyposmic or AG/GG genotype participants. Furthermore, the perception intensity reported by participants showed the following decreasing order: LA > OA > PA. These findings indicate that the intensity and threshold of perception depend on the lipophilicity of the molecule. These results indicate that genetic and biological factors, as well as the chemical properties of the molecules, play a key role in the olfactory perception of fatty acids.

## 1. Introduction

The olfactory system provides individuals with information from both the internal and external environment and one of its main functions is to modulate eating behavior, influencing the duration and composition of a meal and, consequently, body weight, energy balance and health status [1,2,3,4,5,6,7]. Several studies have shown that people with olfactory disorders change their eating habits, preferring foods rich in salt, spices, fats, disaccharides and refined sugars, which are therefore more gratifying, over foods such as fruits and vegetables [3,8,9,10,11,12,13,14,15,16,17].

Depending on environmental factors [18,19,20,21], physiological factors [22,23,24,25], pathological factors [26,27,28,29,30], and genetic factors [31,32,33,34,35], the olfactory function of individuals can vary from normosmia (normal function), to hyposmia (reduced or compromised function) or anosmia (totally or specifically absent function), both for complex stimuli and single molecules [36,37,38,39,40]. Among genetic factors, a key role in olfactory function is played by odorant binding proteins (OBPs) expressed in the perireceptor space of the olfactory epithelium [41,42]. Several studies have suggested that odorants are captured and transported through the mucus layer by OBPs to olfactory receptors (ORs) present in the ciliated ends of the olfactory sensory neurons to facilitate OR/odorant binding [43,44,45,46]. In particular, the human gene encoding OBPIIa presents a single nucleotide polymorphism, *rs2590498* (A/G), which has been associated with significant variations in olfactory sensitivity. Individuals homozygous for the A allele show lower olfactory thresholds and report perceiving odors with higher intensities than individuals who were heterozygous or homozygous for the G allele, indicating a potential functional role of this polymorphism in modulating the olfactory response [42,47,48,49].

Fatty acids commonly found in foods are primarily in the form of triglycerides, but at low concentrations they are also present as free fatty acids [50,51]. Olfactory perception of long-chain fatty acids represents a topic of growing interest in human sensory physiology, as these molecules contribute to the aroma of foods, with implications for the regulation of food intake and in the evaluation of the nutritional quality of foods [52,53]. Recent studies have shown that individuals are also able to perceive the odor of palmitic (C16:0), oleic (C18:1) and linoleic (C18:2) acids through the orthonasal pathway, and that the threshold and intensity of perception vary systematically between individuals and as a function of biological characteristics, such as sex and general olfactory function, as well as the properties of the molecules, such as their lipophilicity [54]. The ability to smell fatty acids from a distance represents an important evolutionary advantage because these molecules play important roles in biological systems in terms of energy reserves, cell membrane composition, thermoregulation, hormone synthesis and absorption of fat-soluble vitamins [55,56,57,58,59,60,61,62].

Given the importance of dietary fatty acids and despite evidence of the involvement of the *rs2590498* polymorphism in general olfactory perception, the specific role of the *OBP* genotype in the perception of long-chain fatty acids has not yet been clarified. Based on these premises, the primary aim of this study was to evaluate the effect of the *rs2590498* (A/G) polymorphism of the human *OBP* gene (OBPIIa) on the threshold and intensity perception of palmitic, oleic, and linoleic acids in healthy individuals. Furthermore, since we had previously found that individual olfactory performance influences fatty acid perception, we investigated the presence of differences in fatty acid perception in individuals classified as normosmic or hyposmic based on their n-butanol threshold. Overall, the proposed integrated approach aims to elucidate the relative contribution of olfactory function and *OBP* polymorphism to the perception of long-chain fatty acids, helping to define the biological and genetic basis of interindividual variability in human olfaction.

## 2. Materials and Methods

### 2.1. Subjects

The volunteers who took part in the study responded to an advertisement published at the University of Cagliari. Based on inclusion criteria (good health, good personal perception of olfactory function, and upper airways not obstructed by allergies or colds), 96 healthy individuals were selected (50 females; 48 males; age 26.8 ± 0.8 years; BMI 18.5–24.99 kg/m^2^). During the recruitment phase, the volunteers were informed of the aim of the study, the experimental procedure, and the commitment time required. Based on this last parameter, all 98 volunteers agreed to participate in the olfactory threshold assessment experiments, while only 86 of them (45 females; 41 males; age 27.2 ± 0.9 years) also took part in the gas chromatography-olfactometer (GC-O) experiments. For all participants, exclusion criteria were the presence of neurodegenerative, tumoral, cognitive, metabolic, inflammatory/autoimmune, cardiovascular and respiratory diseases [6,29,63,64,65,66,67,68,69,70,71,72,73].

All volunteers were asked not to eat, drink (except water), chew gum, and/or smoke for 2 h before the experiment. Each volunteer was required to arrive in the laboratory at least 15 min before the start of the tests to acclimatize to the environment, read the experimental protocol approved by the local ethics committee (Prot. PG/2021/14278, 22 September 2021) and sign an informed consent form.

### 2.2. Evaluation of n-Butanol Odor Threshold

The odor threshold of participants was assessed using the n-butanol threshold test (Thre-test), one of the sub-tests of the Sniffin’ Sticks battery (Burghart Instruments, Wedel, Germany) [74]. The experimenter has 16 triplet pens, each consisting of two blanks filled with a solvent and one target pen containing n-butanol. The pens containing n-butanol have increasing concentrations from triplet 16 (lowest concentration) to triplet 1 (highest concentration). The participant is presented with the triplet with the lowest concentration, and the sequence continues until the pen containing n-butanol is identified twice. This represents the inversion point, or first reversal. The next step is to present triplets containing n-butanol at higher or lower concentrations until seven reversals are achieved. The average of the triplets from the last four reversals represents the n-butanol threshold score of each participant. The score assigned to each participant varies from 1 to 16 and allows them to be classified as normosmic or hyposmic [75].

### 2.3. “Mass Spectrometry-Gas Chromatography-Olfactometry” (MS-GC-O) Technique

A mass spectrometer (MS; Agilent Model 5973; Santa Clara, CA, USA) and an olfactometer (O; Gerstel ODP3; Mülheim a der Ruhr, Germany) were used in conjunction with a gas chromatograph (GC; Agilent 6890N; Santa Clara, CA, USA) for the experiments [36,76]. Ultrapure helium was employed as the carrier gas at a constant flow rate of 1.2 mL/min. After injecting 1 µL of a fatty acid mixture containing 20 mg/mL of palmitic acid (PA), oleic acid (OA), and linoleic acid (LA) into the GC system, the column eluate was split 1:1 ratio between the mass spectrometer and the olfactometer [54]. The mass spectrum found in the NIST2014 library (US National Institute of Standards and Technology; Gaithersburg, MD, USA) was compared with that of volatile compounds.

The chromatographic column employed was an HP-INNOWax (30 m × 0.25 mm × 0.50 µm) (Agilent 19091N-233; Agilent Technologies, Santa Clara, CA, USA). The injector and MS interface temperatures were maintained at 250 °C and 260 °C, respectively. The oven temperature program was set as follows: 40 °C (0.2 min), then increased at 40 °C/min to 100 °C (held for 2 min), followed by a ramp of 10 °C/min to 200 °C (held for 2 min), and finally increased at 10 °C/min to 250 °C (held for 39 min). The chromatographic run lasted 58 min.

Each time the participant perceived the odor of a molecule, using a keyboard digitally connected to the PC (GERSTEL ODP recorder 3 for Windows 7), they provided a personal rating of the perceived odor by pressing one of the 4 keys representing a 4-point intensity scale: 1 = weak odor, 2 = distinct odor, 3 = intense odor, 4 = very intense odor [77,78]. If the participant did not detect a peak, no keys were pressed and a score of “0” was automatically assigned, which was then used for statistical analyses. The PC automatically recorded the retention time and the subjective rating of the perceived odor, allowing the aromatogram to be aligned with the chromatogram [54]. To avoid bias in their perception, the samples were presented blind.

### 2.4. Determination of the Olfactory Threshold of Fatty Acids

The solutions used for each of the fatty acids were chosen on the basis of previous studies and in accordance with their solubility: 0.75, 1.5, 3, 4.5, 6, 9 and 12 mM for palmitic acid (PA); 6, 12, 24, 48, 95, 190, and 380 mM for oleic acid (OA); 0.75, 1.5, 3, 6, 12, 24, and 48 mM for linoleic acid (LA) [54,79]. For data analysis, the seven concentrations of each fatty acid, different due to their different solubilities, were identified as decreasing dilution steps from the highest (marked 1) to the lowest (marked 7). This procedure was chosen to be similar to that of the Thre-test with the n-butanol. The experiment involved seven sets of triplet blotting paper strips (1 × 6 cm). Each set included one strip filled with 20 µL of the fatty acid solution and two strips containing an equal volume of liquid paraffin oil. Participants were asked to identify the strip infused with the fatty acid (target strip). The test begins with the lowest concentration and progresses to higher concentrations until the participant identifies the target strip twice in a row. This is the starting point and represents the first reversal. The test is then decreased until the participant makes an error, and from that point onwards it increases again (second reversal). The sequence of reversals is repeated seven times, and the olfactory threshold is defined as the average of the dilutions of the last four reversals. Each triplet is presented at approximately 20 s intervals. The score assigned to each participant ranges from 1 to 7.

### 2.5. Genetic Analysis

DNA was isolated from 2 mL of unstimulated whole saliva using the standard salting-out procedure. Briefly, samples were centrifuged at 13,000 rpm, and the pellet was resuspended in 500 μL of lysis buffer (1 M NaCl, 0.1 M Tris-HCl pH 8.0, 40 mM EDTA, 0.2% SDS) supplemented with proteinase K (5 μL, 50 mg/mL), followed by 2 h incubation at 56 °C. Proteins were precipitated using saturated sodium acetate (3 M, pH 8.0) and removed by centrifugation at 8000 rpm for 10 min. DNA was precipitated with 100% isopropanol, washed with 70% ethanol, air-dried, and resuspended in 50 μL of nuclease-free water. Genomic DNA was extracted using the QIAamp^®^ DNA Mini Kit (QIAGEN S.r.l., Milan, Italy) according to the manufacturer’s instructions. DNA yield, concentration, and purity were evaluated by measuring absorbance at 260 nm with a NanoDrop One spectrophotometer (Thermo Fisher Scientific, Milan, Italy). Volunteer subjects were genotyped for the *rs2590498* (A/G) polymorphism of the *OBPIIa* gene using a customized TaqMan^®^ SNP Genotyping Assay (code: 4332077, Applied Biosystems by Life Technologies Italia, Milan, Italy Europe BV), as previously described [42,80]. PCR amplification was performed using two primers: a sense (forward) primer (GCCAGGCAGGGACAGA) and an antisense (reverse) primer (CTACACCTGAGACCCCACAAG). Two allele-specific TaqMan fluorescent probes (VIC-labeled probe: TCGGTGACATGAACC and FAM-labeled probe: TCGGTGACGTGAACC) were also used. PCR reactions were carried out in 96-well plates under fast thermal cycling conditions. Each reaction contained 10 ng of DNA, 1× TaqMan^®^ Genotyping Assay (code: 4332077), 1× TaqMan^®^ Genotyping Master Mix (code: 4371355), and nuclease-free water. Amplification and fluorescence detection were performed using the StepOne^TM^ Real-Time PCR System, while genotype determination assignment was achieved by allelic discrimination analysis with Sequence Detection Software (Genotyping module, Applied Biosystems, version 2.3; Life Technologies Italia, Monza, Italy). All samples were analyzed in duplicate, with both positive and negative controls included in each plate run. Molecular analyses revealed that, among participants in the GC-O experiments, 23 subjects were AA homozygous, 16 heterozygous and 40 GG homozygous; meanwhile, among the participants in the experiments aimed at determining the olfactory threshold for fatty acids, we found 25 AA, 28 AG and 45 GG subjects.

### 2.6. Statistical Analysis

One-way MANOVA was used to analyze the effect of n-butanol threshold status (n-butanol T-status) and of the *OBPIIa* genotype on the intensity with which individuals perceived the odors of fatty acids (during elution from the gas chromatographic column) and on the fatty acid detection threshold.

Repeated-measures ANOVA was used to evaluate differences in the perceived intensity of fatty acid odors (during elution from the gas chromatographic column) and in fatty acid detection thresholds.

Data were checked for homogeneity of variance, sphericity and normality. Post hoc comparisons were conducted with Fisher’s least significant difference (LSD) test; Duncan’s test was used in the case that the assumption of homogeneity of variance was violated. *p* values < 0.05 were considered significant. Statistical analysis was performed using STATISTICA for WINDOWS (version 7.0; StatSoft Inc., Tulsa, OK, USA).

## 3. Results

### 3.1. Perceived Intensity of Fatty Acids

**Figure 1:**
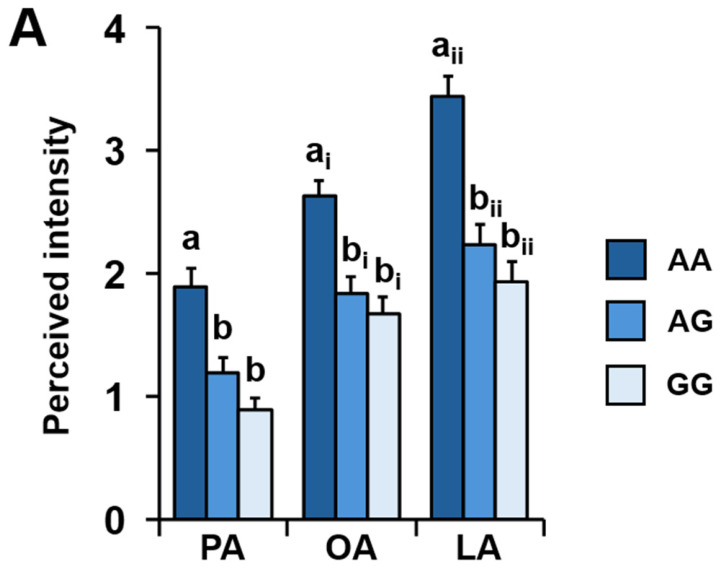

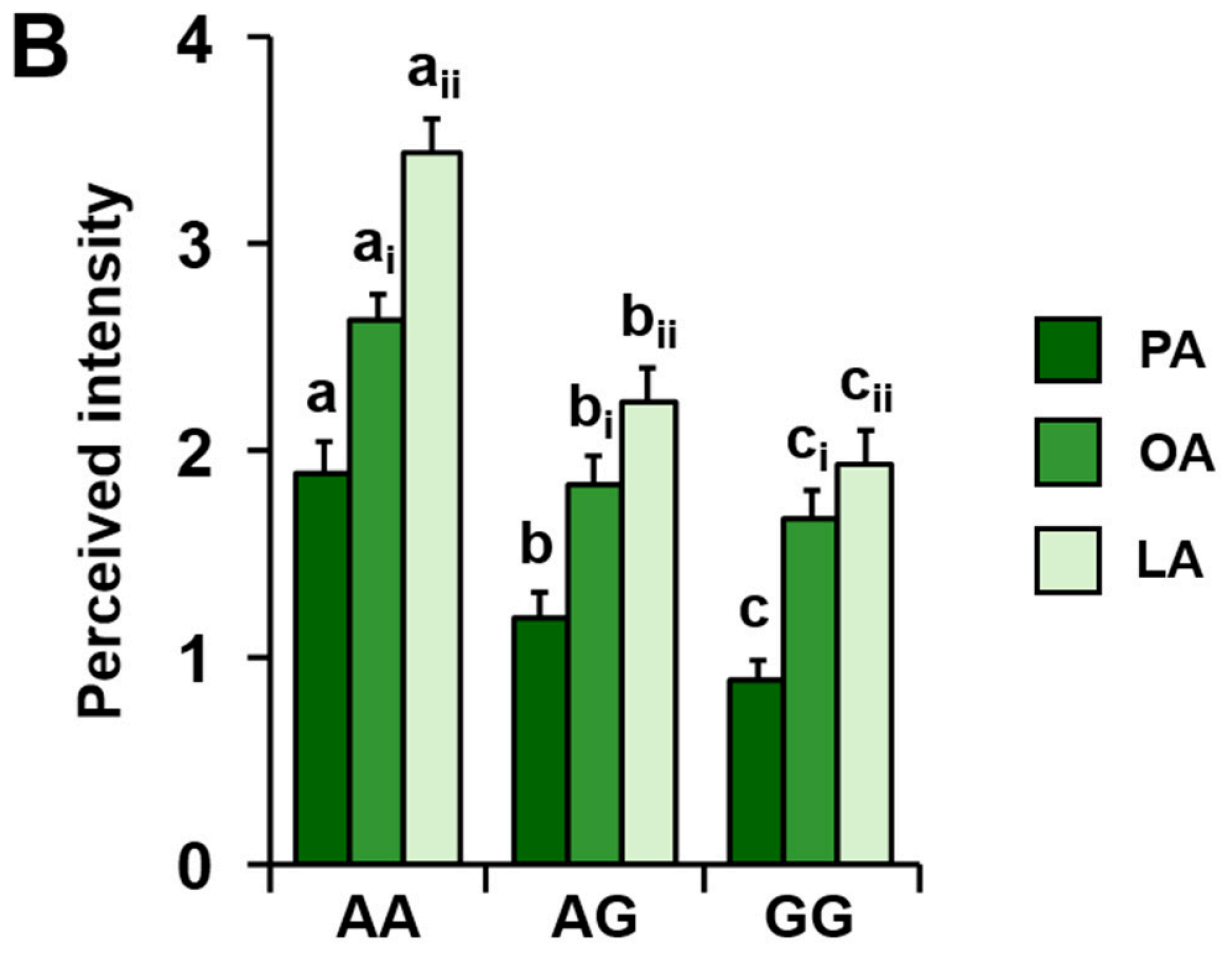
**Effect of the *rs2590498* polymorphism of the *OBPIIa* gene on the perceived intensity of fatty acids.** Mean value ± SE of the intensity perceived for the odors of palmitic (PA), oleic (OA), and linoleic (LA) acids during GC-O experiments, according to the genotypes of the *OBPIIa* locus. (**A**) Different letters indicate significant differences in perceived intensity for the same fatty acid by individuals with different genotypes (PA: a–b, *p* < 0.001; OA: a_i_–b_i_, *p* < 0.001; LA: a_ii_–b_ii_, *p* < 0.001; Fisher’s LSD test following one-way MANOVA). (**B**) Different letters indicate significant differences in perceived intensity for different fatty acids within the same genotype (AA: a–a_ii_, *p* < 0.001; AG: b–b_ii_, *p* < 0.02; GG: c–c_ii_, *p* < 0.05; Fisher’s LSD test following repeated-measures ANOVA).

One-way MANOVA revealed a significant effect of the polymorphism (A/G) of the gene encoding OBP on the perceived intensity of palmitic, oleic, and linoleic acids (F_6,162_ = 7.57; *p* < 0.001; Figure 1A). For each of the three fatty acids, we found that individuals homozygous for A allele perceived the odor with a significantly higher intensity than heterozygous (*p* < 0.001; Fisher’s LSD test) or G homozygous individuals (*p* < 0.001; Fisher’s LSD test). Furthermore, as shown in Figure 1B, repeated-measures ANOVA showed that among individuals with the same genotype, linoleic acid was perceived with the highest intensity, followed by oleic acid and last by palmitic acid (AA: *p* < 0.001; AG: *p* < 0.02; GG: *p* < 0.05; Fisher’s LSD test).

**Figure 2:**
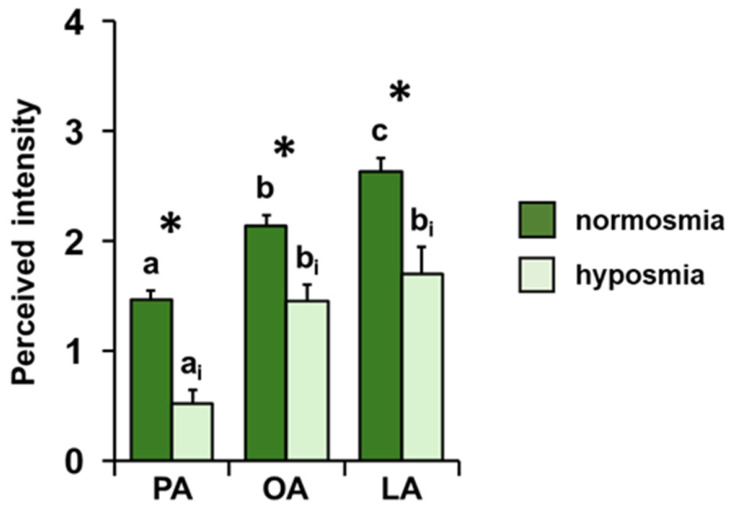
**Effect of individual olfactory status on the perceived intensity of fatty acids.** Mean value ± SE of the intensity perceived for the odors of palmitic (PA), oleic (OA), and linoleic (LA) acids during GC-O experiments, according to olfactory status. (*) indicates significant differences between individuals with normosmia or hyposmia for the same fatty acid (*p* < 0.005; Fisher’s LSD test following one-way MANOVA). Different letters indicate significant differences in perceived intensity for different fatty acids within the same olfactory status (normosmia: a–c, *p* < 0.001; hyposmia: a_i_–b_i_, *p* < 0.001; Fisher’s LSD test following repeated-measures ANOVA).

The mean values ± SE of the intensity with which the odors of palmitic (PA), oleic (OA) and linoleic (LA) acids were perceived by each individual during the GC-O experiments, according to their n-butanol T-status are shown in Figure 2. One-way MANOVA revealed that normosmic individuals perceived the odors of PA (*p* < 0.001; Fisher’s LSD test), OA (*p* < 0.005; Fisher’s LSD test) and LA (*p* < 0.001; Fisher’s LSD test) with a higher intensity than individuals with hyposmia, showing a significant effect of the n-butanol T-status on the perceived intensity for these three fatty acids (F_3,82_ = 10.27; *p* < 0.001). Post hoc analyses following repeated-measures ANOVA (F_2,168_ = 46.264; *p* < 0.001) revealed, both among normosmic and hyposmic individuals for their n-butanol T-status, the presence of a significant increasing order in perceived intensity: PA was perceived with the lowest intensity, followed by OA with an intermediate intensity and finally LA with the highest intensity (PA-OA: *p* < 0.001; OA-LA: *p* < 0.001; Fisher’s LSD test) for normosmic individuals. In addition, the following order of intensity was found for the hyposmic individuals: palmitic < oleic = linoleic (PA-OA: *p* < 0.001; PA-LA: *p* < 0.001; Fisher’s LSD test).

### 3.2. Olfactory Threshold of Fatty Acids

**Figure 3:**
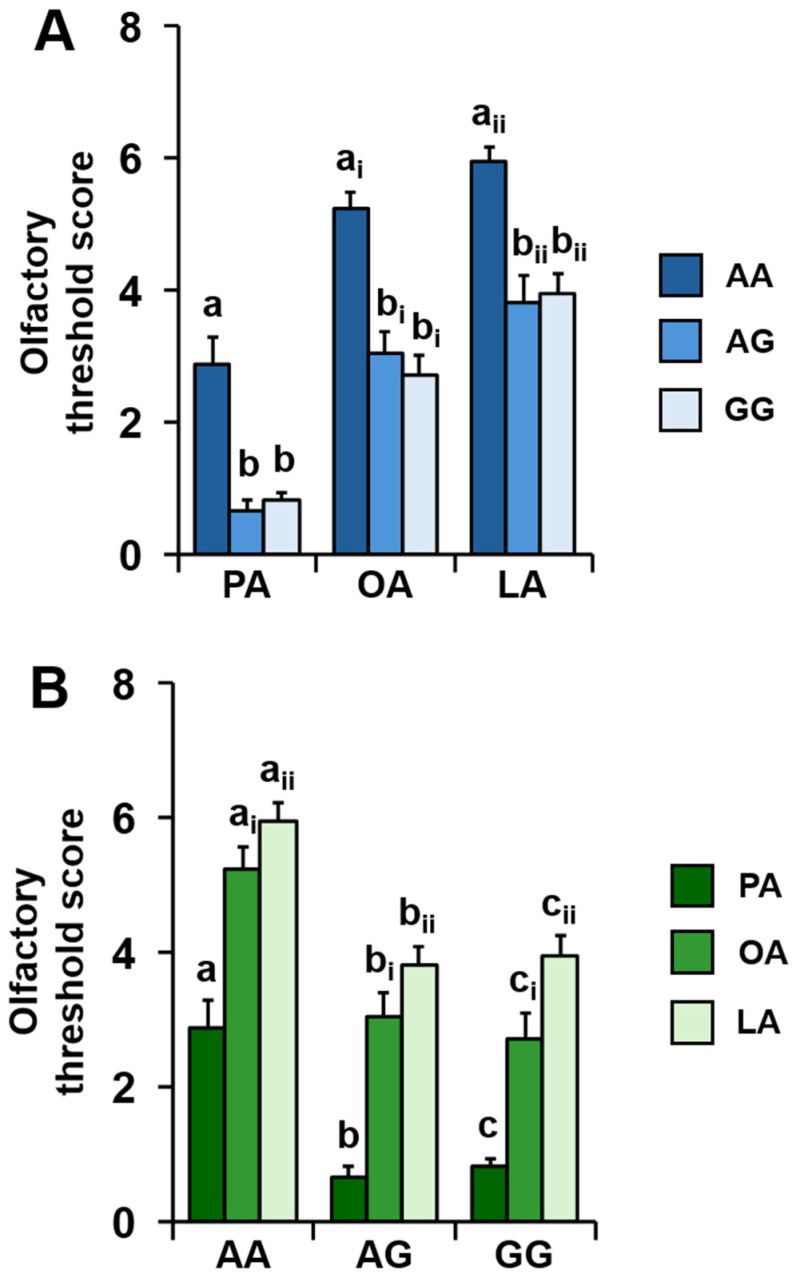
**Effect of the *rs2590498* polymorphism of the *OBPIIa* gene on the olfactory threshold of fatty acids.** Mean value ± SE of the olfactory threshold for the odors of palmitic (PA), oleic (OA), and linoleic (LA) acids during GC-O experiments, according to the genotypes of the *OBPIIa* locus. (**A**) Different letters indicate significant differences in the olfactory threshold for the same fatty acid of individuals with different genotypes (PA: a–b, *p* < 0.001; OA: a_i_–b_i_, *p* < 0.001; LA: a_ii_–b_ii_, *p* < 0.001; Fisher’s LSD test following one-way MANOVA). (**B**) Different letters indicate significant differences in the olfactory threshold for the different fatty acids in individuals within the same genotype (AA: a–a_ii_, *p* < 0.05; AG: b–b_ii_, *p* < 0.05; GG: c–c_ii_, *p* < 0.005; Fisher’s LSD test following repeated-measures ANOVA). The values on the *y*-axis indicate the average score obtained during the threshold test: the higher the score obtained, the lower the olfactory threshold.

One-way MANOVA revealed a significant effect of the polymorphism (A/G) of the gene encoding OBP on the olfactory threshold of palmitic, oleic, and linoleic acids (F_6,186_ = 11.21; *p* < 0.001; Figure 3A). For each of the three fatty acids, we found that in individuals homozygous for A allele, the average score obtained was significantly higher than that obtained by individuals who were heterozygous (*p* < 0.005; Fisher’s LSD test) or G homozygous (*p* < 0.001; Fisher’s LSD test). Furthermore, as shown in Figure 3B, repeated-measures ANOVA showed that among individuals with the same genotype, the olfactory threshold was the lowest for linoleic acid, intermediate for oleic acid and highest for palmitic acid (AA: *p* < 0.05; AG: *p* < 0.05; GG: *p* < 0.005; Fisher’s LSD test).

**Figure 4:**
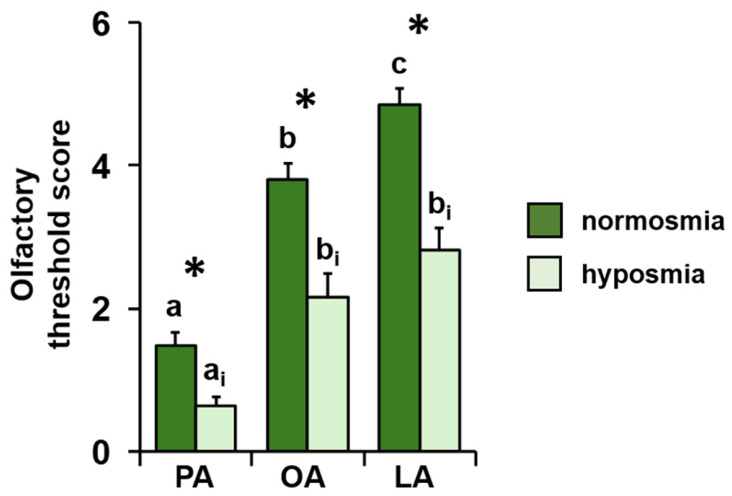
**Effect of individual olfactory status on the olfactory threshold of fatty acids.** Mean value ± SE of the olfactory threshold for the odors of palmitic (PA), oleic (OA), and linoleic (LA) acids during GC-O experiments, according to their olfactory status. (*) indicates significant differences between individuals with normosmia or hyposmia for the same fatty acid (*p* < 0.05; Fisher’s LSD test following one-way MANOVA). Different letters indicate significant differences in the olfactory threshold for the different fatty acids within the same olfactory status (normosmia: a–c, *p* < 0.001; hyposmia: a_i_–b_i_, *p* < 0.001; Fisher’s LSD test following repeated-measures ANOVA). The values on the *y*-axis indicate the average score obtained during the threshold test: the higher the score obtained, the lower the olfactory threshold.

The mean values ± SE of the olfactory threshold of participants for the odors of palmitic (PA), oleic (OA) and linoleic (LA) acids, according to their n-butanol T-status, are shown in Figure 4. One-way MANOVA revealed a significant effect of the n-butanol T-status on the olfactory threshold of participants for fatty acids (F_3,94_ = 7.42; *p* < 0.001). Compared to participants with hyposmia, normosmic individuals showed lower thresholds for linoleic acid (*p* < 0.001; Fisher’s LSD test), intermediate thresholds for oleic acid (*p* < 0.001; Fisher’s LSD test), and higher thresholds for palmitic acid (*p* < 0.03; Fisher’s LSD test). In fact, they reached the highest score for LA, intermediate score for OA, and lowest score for PA. Repeated-measures ANOVA revealed, among both normosmic and hyposmic individuals for their n-butanol T-status, the presence of a significant decreasing order in their fatty acid threshold (F_2,192_ = 3.24; *p* < 0.05). In particular, post hoc analyses showed that, in normosmic participants, the score obtained was lower for PA, intermediate for OA and higher for LA (PA-OA: *p* < 0.001; OA-LA: *p* < 0.001; Fisher’s LSD test); meanwhile, for hyposmic subjects the following order of threshold score was found: palmitic < oleic = linoleic (PA-OA: *p* < 0.001; PA-LA: *p* < 0.001; Fisher’s LSD test).

## 4. Discussion

The intensity with which odors are perceived influences eating behavior, not only in terms of food choices, but also in terms of quantity and duration of the meal. In fact, it has been shown that the greater the intensity of the perceived odor of a food, the more quickly sensory satiety is reached for that food, determining the end of its ingestion [81,82,83]. In the case of high energy/calorie foods, such as fatty ones, this is very important because it could prevent the ingestion of large quantities of potentially harmful foods. A diet rich in high-calorie foods is one of the main causes of obesity [84], which in turn is associated with a pro-inflammatory state and cardiovascular diseases [85,86,87]. Individuals with better olfactory function and the ability to sensorially evaluate food composition, such as the fatty acids present in lipid-rich foods, could have an evolutionary advantage because they are able to more effectively assess food composition and its caloric/nutritional content.

Among the factors involved in the interindividual variability of olfactory function, a significant role appears to be played by the different expression and function of OBPs in the perireceptor space, a thin layer of water and mucus that bathes the ciliated ends of the olfactory sensory neurons (OSNs) of the olfactory epithelium. OBPs would have the role of capturing and transporting odorants, mainly of lipophilic nature, to the ORs and facilitating their binding [41,42,43,88,89,90,91,92]. It has been previously shown that both the intensity with which the odor intensity of palmitic, oleic and linoleic acids is perceived, as well as their perception threshold, vary according to the number of double bonds present in the molecule; in particular, linoleic acid (polyunsaturated) has the lowest perception threshold and the highest intensity, oleic acid (unsaturated) has an intermediate perception threshold and intensity, and palmitic acid (saturated) has the highest perception threshold and the lowest intensity [54]. Since their perception is inversely correlated with the lipophilicity of the fatty acid molecule, the first aim of this study was to investigate the role of the *rs2590498* polymorphism of the human gene encoding OBPs both in the perception threshold and the intensity with which palmitic, oleic and linoleic acid molecules are perceived by individuals. The results we obtained show that individuals carrying two A alleles perceive the odor of palmitic, oleic and linoleic acids with a higher intensity and have a lower perception threshold than individuals with heterozygous or GG homozygous alleles. These results are consistent with previous studies showing that individuals homozygous for the major A allele perceive simple and complex odors with greater intensity than individuals with the other two genotypes [47,48,49]. Furthermore, we found that the intensity of perception is directly proportional to the number of double bonds present in the molecule, showing the following decreasing order, LA > OA > PA for AA homozygous, heterozygous, and GG homozygous participants. Consequently, the perception threshold is inversely proportional to the number of double bonds: the lowest threshold for linoleic acid (polyunsaturated), intermediate for oleic acid (unsaturated) and highest for palmitic acid (saturated). These findings highlight that the water solubility of molecules and the OBPs genotype are central factors in their perception. Indeed, individuals with AA genotype are advantaged by the presence not only of a greater number of OBPs, but also of more functional ones; these, therefore, bind odorants more easily and a greater number of molecules interact with the ORs, activating a more intense signal transduction and coding cascade [88,93]. Linoleic acid, being the most water soluble due to the number of double bonds, would be more easily captured by OBPs and a greater number of molecules would be transported to the ORs, making their binding and OSN activation more likely [94]. This would explain why even individuals with at least one G allele, and therefore with fewer and less functional OBPs, perceive linoleic acid more intensely than oleic acid, and the latter more intensely than palmitic acid.

Previous studies have shown that the olfactory threshold of individuals is at least partially determined by the *rs2590498* polymorphism of the gene encoding OBPIIa, the only one present in the human olfactory epithelium [41,42]. Given the relationship we found between the OBPs genotype and fatty acid perception, the second aim of our work was to study the ability to perceive palmitic, oleic and linoleic acids in individuals classified as normosmic or hyposmic on the basis of their n-butanol T-status. The results we obtained show that normosmic individuals perceive the odor of all fatty acids considered more intensely than hyposmic individuals and also show a lower perception threshold (as indicated by the higher score obtained). In addition, for both categories of individuals, we observed that perception is directly proportional to the solubility of the molecules in water. Specifically, we found the following decreasing order of perceived intensity: PA < OA < LA for normosmic individuals and PA < OA = LA for individuals with hyposmia. Regarding the perception threshold, we observed the following decreasing order: PA > OA > LA for normosmic individuals and PA > OA = LA for participants with hyposmia. In accordance with this, a relationship between the perception of fatty acids and the general olfactory function of individuals has previously been found [54]. Furthermore, this result is consistent with the finding that *OBP* genotype influences fatty acid perception, both in terms of intensity and threshold. It should be remembered that the degree of activation of OSNs at the peripheral level determines the intensity with which sensory information reaches the CNS and, therefore, the intensity with which a molecule is perceived. The fact that linoleic acid has the lowest perception threshold is consistent with its reduced lipophilicity: it is captured and transported more easily by OBPs and is therefore perceived with greater intensity. Conversely, palmitic acid is perceived with less intensity because, being the most lipophilic, it is less easily captured by OBPs and, consequently, has a higher threshold and a lower perception intensity.

This aspect is very important in relation to the role that smell plays in the eating behavior of individuals [3,5,8,13,14]. Individuals with normosmia, having better olfactory function, appear to be able to better evaluate food composition and choose healthier and more functional foods. This hypothesis is supported by a previous study in which individuals with normosmia show greater adherence to the Mediterranean diet, characterized by a high consumption of fruits, vegetables, and monounsaturated fats and a reduced consumption of processed meat, refined sugars, and saturated fats [13,87,88,89,90]. Similarly, individuals with AA genotype, who show better olfactory function, may exhibit eating behavior similar to that of individuals with normal olfactory function, although further studies will be needed to test this hypothesis. However, this aspect could be partially compensated by the properties of food. Highly hydrophilic foods counteract reduced olfactory function because they move more easily in the perireceptor space and, therefore, have an equal opportunity to reach the ORs and activate the transduction and coding cascade that characterizes the receptor response. Conversely, highly lipophilic foods, when associated with reduced olfactory function, could be more easily chosen and pose a risk to human health. In fact, while palmitic acid (saturated) has been associated with cardiovascular risk, high blood levels of LDL cholesterol, insulin resistance and the onset of diabetes mellitus, oleic (unsaturated) and linoleic (polyunsaturated) acids, on the contrary, appear to reduce blood pressure, LDL cholesterol levels, inflammatory status and insulin resistance [55,56,57,58,60,61,62,95,96,97,98]. The lower lipophilicity of linoleic and oleic acid could therefore also represent an advantage for hyposmic individuals or those carrying at least one G allele, as it could allow them to more accurately evaluate the caloric content of foods and choose foods that are both calorie rich and healthy. This advantage is particularly important for linoleic acid which, as an essential fatty acid, cannot be synthesized in sufficient quantities through metabolic pathways and must be obtained through the diet [56].

## 5. Conclusions

The results of this study show that fatty acid perception is influenced by genetic factors (polymorphism of the *OBPs* gene), biological factors (individual odor threshold), and the chemical properties of the molecules (water solubility). Individuals with AA genotype, compared to those with at least one G allele, possess a greater number of functional OBPs and, consequently, have better olfactory function. We suggest that these individuals may perceive the odor of palmitic, oleic, and linoleic acids more intensely and at lower concentrations, facilitating the choice of foods rich in essential fatty acids and low in calories. Furthermore, the number of double bonds, which make the molecules more water-soluble and favor their interaction with OBPs, may also represent an advantage for individuals with AG or GG genotypes.

## Data Availability

The data presented in this study are available on request from the corresponding author. The data is not publicly available due to restrictions (e.g., privacy or ethical).
